# Proteolysis of the low‐density lipoprotein receptor in hepatocytes is mediated by BMP1 but not by other astacin proteases

**DOI:** 10.1002/1873-3468.14667

**Published:** 2023-06-05

**Authors:** Katherine A. B. Kellett, Kate Fisher, Harry Aldworth, Nigel M. Hooper

**Affiliations:** ^1^ Division of Neuroscience, School of Biological Sciences, Faculty of Biology, Medicine and Health University of Manchester UK; ^2^ Present address: Horizons Institute University of Leeds Leeds LS2 9JT UK

**Keywords:** astacin, BMP1, hepatocytes, LDL receptor, LDL‐cholesterol, meprin, protease

## Abstract

Bone morphogenetic protein 1 (BMP1), a member of the astacin family of zinc‐metalloproteases, proteolytically cleaves the low‐density lipoprotein receptor (LDLR) within its ligand‐binding domain, reducing the binding and cellular uptake of LDL‐cholesterol. Here, we aimed to determine whether astacin proteases other than BMP1 may also cleave LDLR. Although human hepatocytes express all six astacin proteases, including the meprins and mammalian tolloid, we found through pharmacological inhibition and genetic knockdown that only BMP1 contributed to the cleavage of LDLR in its ligand‐binding domain. We also found that the minimum amino acid change required to render mouse LDLR susceptible to cleavage by BMP1 is mutation at the P1′ and P2 positions of the cleavage site. When expressed in cells, the resulting humanised‐mouse LDLR internalised LDL‐cholesterol. This work provides insight into the biological mechanisms regulating LDLR function.

## Graphical abstract

The low‐density lipoprotein receptor (LDLR) binds LDL‐cholesterol at the cell surface and internalises. The protease BMP1 cleaves LDLR in its ligand‐binding domain, preventing the binding and internalisation of LDL‐cholesterol. BMP1 is a member of the astacin family of zinc metalloproteases, however, no other astacin protease, including the meprins, is involved in cleaving LDLR in human hepatocytes.

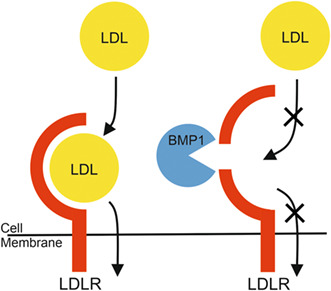

## Abbreviations

**BMP1**, bone morphogenetic protein 1

**BODIPY‐LDL**, [boron dipyrromethene (4,4‐difluoro‐4‐bora‐3a,4a‐diaza‐s‐indacene)]‐LDL

**CTF**, C‐terminal fragment

**CUB**, complement C1r/C1s, Uegf, BMP1

**EGF**, Ca^2+^‐binding epidermal growth factor‐like

**FBS**, fetal bovine serum

**FL**, full length

**HEK**, human embryonic kidney

**hLDLR**, human low density lipoprotein receptor

**h‐mLDLR**, humanised mouse low density lipoprotein receptor

**LA repeats**, low density lipoprotein receptor type A repeats

**LDL‐C**, low density lipoprotein cholesterol

**LDLR**, low density lipoprotein receptor

**mep α**, meprin α

**mep β**, meprin β

**mLDLR**, mouse low density lipoprotein receptor

**mTLD**, mammalian tolloid

**NTF**, N‐terminal fragment

**PBS**, phosphate‐buffered saline

**PBS‐T**, phosphate‐buffered saline containing 0.1% (v/v) Tween

**TLL1**, tolloid‐like protein 1

**TLL2**, tolloid‐like protein 2

Cardiovascular disease is intimately linked to elevated plasma levels of low‐density lipoprotein cholesterol (LDL‐C) in the blood. The amount of functional low‐density lipoprotein receptor (LDLR) expressed on the surface of hepatocytes is the primary determinant of plasma LDL‐C [1]. LDLR binds plasma LDL‐C and the complex is then internalised *via* clathrin‐mediated endocytosis. In the acidic environment of the endosome, the LDL‐C dissociates from the receptor which is recycled to the cell surface, while the LDL‐C is delivered to lysosomes for degradation. The activity of LDLR is tightly regulated through its biosynthesis, surface localisation, internalisation, recycling and degradation in order to maintain plasma cholesterol homeostasis (reviewed in [2]). Recently, the zinc metalloprotease bone morphogenetic protein 1 (BMP1; also known as procollagen C‐peptidase) was identified in two independent studies as selectively cleaving LDLR within its ligand binding repeats, reducing the binding and cellular uptake of LDL‐C [3, 4].

Human LDLR is an 860 amino acid type 1 transmembrane glycoprotein consisting of multiple domains. The ligand‐binding domain (residues 22–314) consists of seven LDLR type A (LA) repeats each of which is ~ 40 amino acids in length. Each of the LA repeats is connected to the next with a small linker of 4–5 residues, with the exception of the linker between LA repeats 4 and 5 which is 12 amino acids long. Binding of LDL‐C to LDLR requires LA repeats 3 through 7 [5, 6]. LDLR is cleaved at the peptide bond between Gly192 and Asp193 in the linker region between the fourth and fifth ligand‐binding repeats (human (h)LDLR numbering including the signal peptide; note that this was previously cited as Gly171‐Asp172 in the mature protein without the signal peptide) [3]. This proteolytic cleavage results in the release of a 36–40 kDa N‐terminal fragment (NTF) of LDLR into the extracellular media and the retention of a 120 kDa C‐terminal fragment (CTF) in the cell membrane.

Using an *in silico* bioinformatics approach we identified BMP1 as a candidate protease for cleaving LDLR at the Gly192‐Asp193 peptide bond. In a cell‐free assay recombinant BMP1 cleaved recombinant human LDLR at the Gly192‐Asp193 bond as determined by N‐terminal protein sequencing, indicating that BMP1 cleaves LDLR at the physiologically relevant site in the ligand binding repeats [3]. The cleavage of LDLR by BMP1 was inhibited by a selective active site‐directed small molecule BMP1 inhibitor, UK383367 [3, 7]. The 120 kDa CTF generated by proteolytic cleavage of LDLR by BMP1 had a reduced ability to bind LDL‐C, and when expressed in LDLR‐deficient CHO‐A7 cells the 120 kDa CTF had a reduced capacity to endocytose LDL‐C compared to cells expressing the full‐length receptor [3]. Genetic knockdown or pharmacological inhibition of BMP1 in HepG2 cells decreased the proteolytic cleavage of LDLR and increased the cellular uptake of LDL‐C [3]. Together these data indicate that BMP1 regulates cellular LDL‐C uptake through proteolytic cleavage of LDLR in its ligand‐binding domain.

Asp at the P1′ position of its substrates is critical for cleavage by BMP1 [8]. Interestingly Asp193 in human LDLR (hLDLR) is not conserved in mouse LDLR (mLDLR) but replaced by Val. Recombinant mLDLR was not cleaved by recombinant BMP1 and only the full length 160 kDa form of the receptor was observed in the liver lysates of wild type mice [3]. Substitution of Asp193 in hLDLR with Val prevented its cleavage when expressed in HepG2 cells [3], confirming the critical requirement for an Asp residue at the P1′ position for cleavage by BMP1. However, whether substituting Val for Asp in mLDLR will render the protein sensitive to cleavage by BMP1 is not known.

**Fig. 1:**
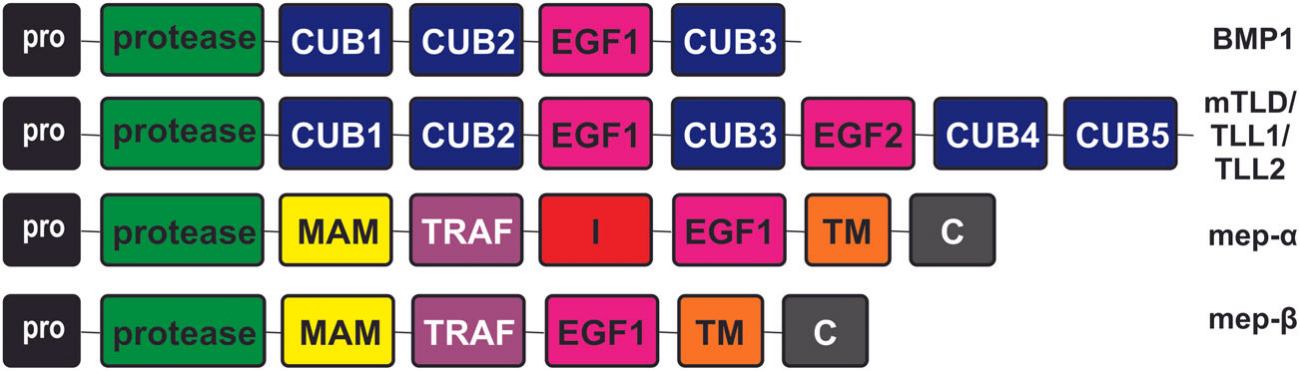
A comparison of the astacin proteases. Schematic diagram of the domain organisation of the astacin proteases showing the similarity and differences between the family members. BMP1, bone morphogenetic protein 1; mep‐α, meprin α; mep‐β, meprin β; mTLD, mammalian tolloid; TLL1, tolloid‐like protein 1; TLL2, tolloid‐like protein 2. Domain abbreviations: C, cytosolic; CUB, complement C1r/C1s, Uegf, BMP1; EGF, Ca^2+^‐epidermal growth factor‐like; I, inserted; MAM, meprin, A5 protein, and receptor protein tyrosine phosphatase μ; Pro, pro‐peptide; protease, zinc‐binding active site; TM, transmembrane; TRAF, tumour necrosis factor receptor‐associated factor.

BMP1 is a member of the astacin family of zinc metalloproteases [9]. In the human and mouse genomes there are six astacin family genes (meprin α, meprin β, BMP1 and its major splice variant mammalian tolloid (mTLD), mammalian tolloid‐like proteins 1 and 2 (TLL1 and TLL2) and ovastacin which is only found in oocytes; Fig. 1). They are structurally related proteases containing a conserved zinc binding active site domain and multiple protein–protein interaction domains – CUB (Complement C1r/C1s, Uegf, BMP1), EGF (Ca^2+^‐binding epidermal growth factor‐like), MAM (meprin, A5 protein, and receptor protein tyrosine phosphatase μ) and TRAF (tumour necrosis factor receptor‐associated factor) [9] (Fig. 1). Both meprin α and meprin β have transmembrane domains towards their C‐terminus, while BMP1, mTLD, TLL1 and TLL2 are secreted, extracellular proteases (Fig. 1).

Given the catalytic and structural similarities between the astacin family members and thus the potential for overlapping substrate specificity, the first aim of this study was to determine whether members of the astacin family, other than BMP1, are involved in the proteolytic cleavage of LDLR in human hepatocytes. Pharmacological inhibition and genetic knockdown were used to assess the contribution of each of the astacin proteases to the cleavage of LDLR within its ligand‐binding domain. A further aim was to explore whether amino acid substitutions in the linker region between the fourth and fifth LA repeats in mLDLR would render it susceptible to cleavage by BMP1. Selective mutagenesis was used to identify the minimum amino acid substitutions required to render mLDLR susceptible to cleavage by BMP1.

## Materials and methods

### Cell culture

Human hepatocellular carcinoma (HepG2) cells were maintained in Advanced Minimum Essential Medium (Advanced‐MEM; Invitrogen Life Technologies, Carlsbad, CA, USA) containing 10% (v/v) foetal bovine serum (FBS; Biosera, Uckfield, UK) and 2 mm l‐glutamine (Gibco, Life Technologies, Paisley, UK). Human adrenal carcinoma (SW13) and human embryonic kidney (HEK) 293 cells were maintained in Dulbecco's modified Eagle's Medium (Lonza, Basel, Switzerland) containing 10% (v/v) FBS. Chinese hamster ovary cells lacking LDLR (CHO‐A7) were obtained from Professor N. G. Seidah (University of Montreal, QC, Canada) and maintained in Ham's F12 medium containing l‐Glutamine (Lonza) and supplemented with 10% (v/v) FBS. HepG2, SW13 and CHO‐A7 cells were passaged using trypsin EDTA (Lonza). Assay‐ready expanded hepatocytes (Axol #A3704, Axol Bioscience, Easter Bush, UK) were revived using hepatocyte thawing medium (Axol) and maintained using hepatocyte maintenance medium (Axol). All cell lines were cultured in a humidified atmosphere of 5% CO_2_ at 37 °C.

### Cell lysate and conditioned media preparation

Conditioned media samples were concentrated to 200 μL using 10 kDa cut‐off Vivaspin filtration columns (Millipore, Billerica, MA, USA). Cells were washed twice in phosphate‐buffered saline (PBS), harvested and pelleted by centrifugation. Cell pellets were lysed, on ice, in 50 mm Tris/HCl, 150 mm NaCl, 0.5% (w/v) sodium deoxycholate, 1% (v/v) Nonidet P‐40, (pH 8.0) with protease inhibitor cocktail. Lysates were clarified by centrifugation at 13 000 ***g*** for 10 min. The protein concentration of the samples was determined using a bicinchoninic acid assay (Sigma‐Aldrich, St Louis, MO, USA).

### Cell surface biotinylation and pulldown of biotinylated proteins

HepG2 cells were grown to 80% confluency and incubated in OptiMEM with or without 10 μm UK383367 for 18 h. Cells were washed with ice‐cold PBS and incubated in PBS with 800 μm EZ‐Link Sulfo‐NHS‐SS‐Biotin (Thermoscientific, Rockford, IL, USA) for 1 h at 4 °C. Cells were washed in PBS with 100 mm glycine to quench biotinylation, harvested and lysed. Lysates (500 μg total protein) were loaded onto 80 μL NeutrAvidin Agarose Resin (Thermoscientific) and incubated for 3 h at 4 °C. The NeutrAvidin Resin was washed three times with lysis buffer and bound proteins were eluted in 2× dissociation buffer (160 mm Tris–HCl, 4% (w/v) SDS, 200 mm dithiothreitol, 20% (v/v) glycerol, 0.1% (w/v) Coomassie Brilliant Blue, pH 6.8) and heated to 95 °C for 5 min. Eluted proteins were analysed by SDS/PAGE and western blotting.

### SDS/PAGE and western blotting

Samples were resolved on either 8% (for full‐length LDLR and CTF) or 12% (for NTF) polyacrylamide gels and transferred to Hybond‐P polyvinylidene difluoride membrane (GE Healthcare, Little Chalfont, Buckinghamshire, UK). The membranes were then blocked for 1 h in PBS containing 0.1% (v/v) Tween (PBS‐T) and 5% (w/v) dried milk powder at room temperature. Membranes were incubated overnight at 4 °C in primary antibody diluted in 2% BSA in PBS‐T: goat anti‐human LDLR (AF2148), goat anti‐mouse LDLR (AF2255; R&D Systems, Minneapolis, MN, USA), chicken anti‐LDLR (Ab14056; Abcam, Cambridge, UK) at a dilution of 1 : 1000, and anti‐actin antibody (Sigma‐Aldrich) at a dilution of 1 : 10 000. Membranes were washed in PBS‐T and then incubated with the appropriate peroxidase‐conjugated secondary antibody, rabbit anti‐goat, rabbit anti‐chicken or rabbit anti‐mouse (Sigma‐Aldrich) at a 1 : 4000 dilution in 2% BSA in PBS‐T. Membranes were washed in PBS‐T with a final wash in PBS before detection by the enhanced chemiluminescence detection method using Pierce Enhanced Chemiluminescence Western Blotting Substrate (Fisher Thermo Scientific, Cramlington, Northumberland, UK). Blots were developed using a Syngene Gbox XX6. Alterations in contrast were made across the whole blot before the analysis of blot densitometry using genetools software (Syngene, Cambridge, UK). Blot images were processed using CorelDRAW graphics and images cropped to highlight bands of interest for clarity.

### qPCR

Hepatocytes, HepG2, SW13 and HEK cells were harvested and RNA was extracted using the RNeasy Plus Kit (QIAgen, Hilden, Germany) according to the manufacturer's instructions. cDNA was synthesised according to the manufacturer's instructions, using 300–1000 ng of prepared RNA using the iScript cDNA Synthesis Kit (BioRad, Watford, UK). BMP1, mTLD, meprin α, meprin β, TLL1 or TLL2 mRNA expression levels were then quantified using real‐time qPCR using the SYBR green method (Applied Biosystems, Life Technologies, Warrington, UK). The following primer sequences were used:

BMP1: forward primer 5′‐TCGTTCGTGAGAACATCCAGC‐3′ and reverse primer 5′‐CTGTCGAAGTCATAGGTCTCC‐3′.

mTLD: forward primer 5′‐ACTCAGATAACTCGGTCCAGC‐3′ and reverse primer 5′‐TAGTTGTTGTCGCCAAACTGG‐3′.

meprin α: forward primer 5′‐ATTTCAACAGTTTGATGGGTGCT‐3′ and reverse primer 5′‐ATGGCCTTATAGGCACATCCT‐3′.

meprin β: forward primer 5′‐AGAGTGATCACTCCAACATGG‐3′ and reverse primer 5′‐TACTTTCCAGCACTGCTGTGG‐3′.

TLL1: forward primer 5′‐GGAAAGAATATGGCCTGGAGG‐3′ and reverse primer 5′‐CATGTGTGCTTTTCCCAGTGC‐3′.

TLL2: forward primer 5′‐AGTCATCCCCTACGTCATTGG‐3′ and reverse primer 5′‐ATGAAGGTCACACAGGTGTGC‐3′.

Relative mRNA expression of each target gene was evaluated using quantitative real‐time RT‐PCR using the ABI 7900HT fast real‐time PCR system. Relative expression was calculated from the standard curve and normalised to ribosomal mRNA expression (RPL13A) as a loading control.

### Inhibitor treatments

HepG2 cells and hepatocytes were grown to 60–70% confluency in 6‐well plates before treatment with inhibitors. Cells were incubated in 2.5 mL of Opti‐MEM + GlutaMAX (Gibco) with or without the addition of UK383367 (R&D Systems), GM6001 (Sigma) or actinonin (Sigma) all at 10 μm for 18 h before cells and the conditioned cell medium were harvested.

### siRNA knockdown

siRNA against human BMP1 (assay ID: s501080, ThermoFisher), mTLD (assay ID: s501082, ThermoFisher), meprin α (assay ID: s230359, Ambion, Life Technologies, Paisley, UK), TLL1 (assay ID: s14179, Ambion), TLL2 (ON‐TARGETplus SMARTpool) and a non‐targeting sequence (Dharmacon, Lafayette, CO, USA) were used in HepG2 cells and hepatocytes. siRNA (50 nm) was delivered as a complex with DharmaFECT‐1 transfection reagent (Dharmacon) according to the manufacturer's protocol. Cells were incubated for 48 h for siRNA knockdown, washed and then further incubated for 24 h in Opti‐MEM before harvesting cells and conditioned media for western blotting and qPCR analysis.

### Plasmids, site‐directed mutagenesis and transient transfection

cDNA encoding either the full‐length human LDLR or mouse LDLR with a 5′ signal peptide sequence and a 3′ FLAG tag were inserted into the mammalian expression vector pcDNA3.1(+) within the 5′ Hind III and 3′ Bam HI sites. Site‐directed mutagenesis was performed on mLDLR to create the K192Q, V194D and double mutation construct, termed h‐mLDLR. HepG2 or CHO‐A7 cells were transfected with the indicated LDLR using the *Trans*IT‐LT1 transfection reagent (Mirus, Madison, WI, USA) according to the manufacturer's protocol. Cells were incubated for 24 h before the medium was replaced with Opti‐MEM for a further 24 h before being harvested for western blot analysis or incubated for 48 h before being fixed for immunofluorescence microscopy. For BMP1 inhibitor experiments, 10 μm UK383367 was added after 24 h for a further 18 h incubation before harvesting.

### BODIPY‐LDL uptake assay

CHO‐A7 cells were seeded onto clear bottomed, black walled 96‐well plates in cell culture medium before transfection with LDLR constructs (mLDLR, h‐mLDLR or hLDLR) for 36 h. Cells were then incubated for a further 18 h incubation in serum‐free culture media. Medium was then replaced with serum‐free culture medium containing 10 μg·mL^−1^ BODIPY [boron dipyrromethene (4,4‐difluoro‐4‐bora‐3a,4a‐diaza‐s‐indacene)]‐LDL (ThermoFisher) and cells were incubated for 6 h. To determine cellular BODIPY‐LDL uptake the media was removed, the cells were washed twice in PBS with a final volume of 50 μL PBS added before measuring the fluorescence in a Synergy HT plate reader using excitation/emission wavelengths of 485 nm/535 nm.

### Statistical analysis

The data were analysed using graphpad prism version 8.0 (GraphPad Software Inc., San Diego, CA, USA). Details of statistical analyses for each data set are described in the relevant figure legends.

## Results

### LDLR is present as the full‐length and proteolytically cleaved forms in human hepatocytes

**Fig. 2:**
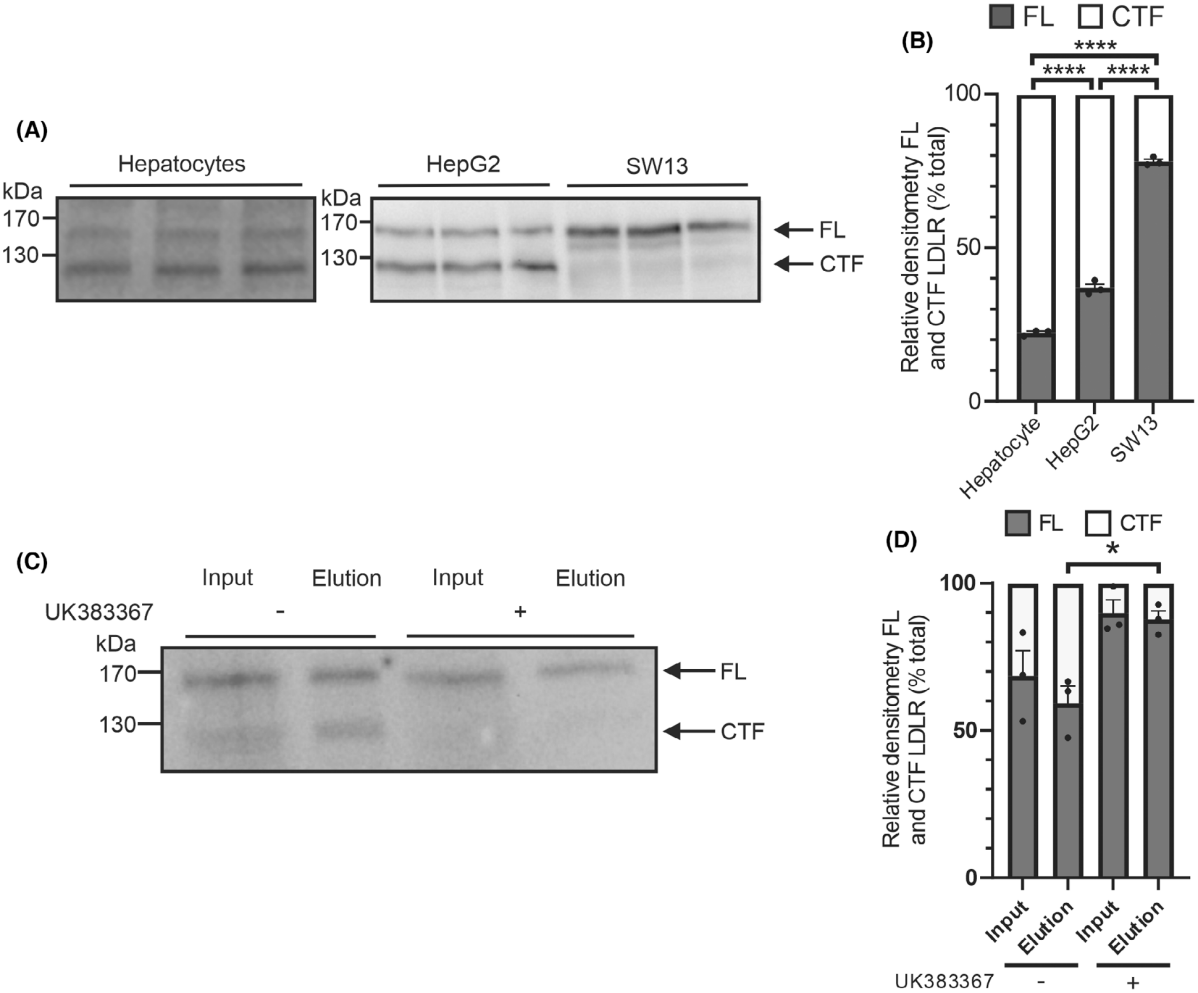
The LDLR profile and cell surface distribution of the CTF in human cells. (A) The LDLR profile in lysates from human hepatocytes, HepG2 and SW13 cells was compared by immunoblot of cell lysates using the LDLR antibody AF2148. (B) The relative amounts of the 170 kDa full‐length (FL) LDLR and the 130 kDa CTF were quantified by densitometry and expressed as a percentage of the total LDLR (FL + CTF). Cell surface biotinylation was performed on HepG2 cells treated with or without UK383367, and biotinylated proteins isolated from lysates *via* pulldown with NeutrAvidin agarose resin. (C) The LDLR profile in input and elution samples from neutravidin pulldown performed on cell lysates. (D) The relative amounts of the biotinylated FL and CTF LDLR were quantified by densitometry and expressed as a percentage of the total LDLR. Data shown as mean ± SEM; LDLR FL to CTF comparisons were made using a 2‐way ANOVA with Dunnett's multiple comparison test to compare the groups. Biotinylated LDLR FL to CTF comparisons were made using an ordinary one‐way ANOVA test with Tukey's multiple comparison test to compare between groups. *P < 0.05, ***P < 0.001, ****P < 0.0001, *n* = 3 for all analyses.

Initially, the presence of the CTF of LDLR in human primary hepatocytes was investigated and compared with that in HepG2 hepatocyte carcinoma cells in which LDLR is proteolytically cleaved in its ligand‐binding domain by BMP1 [3] (Fig. 2A). In lysate from the primary hepatocytes, LDLR appeared as the 170 kDa full‐length protein and the 130 kDa CTF. The ratio of the CTF to the full‐length LDLR was significantly greater in the hepatocytes than in the HepG2 cells (Fig. 2B). For comparison, in human adrenal SW13 cells there was a minimal amount of LDLR cleavage (Fig. 2A,B), consistent with our previous observation [3]. To clarify whether the 130 kDa band was the cleaved form of full‐length cell surface LDLR or an intracellular precursor form of the receptor, cells were incubated with a cell‐impermeable biotinylation reagent in the absence or presence of the BMP1 inhibitor UK383367. There was a similar amount of the 130 kDa CTF present in the total cell input sample as in the cell surface biotinylated sample (Fig. 2C,D). In the presence of the BMP1 inhibitor there was a similar reduction in the amount of CTF in both the total cell input sample and the cell surface biotinylated sample, with nearly all the 130 kDa CTF band in both the input and elution samples disappearing (Fig. 2C,D), indicating that this band predominantly consists of the CTF cleaved from full‐length LDLR by BMP1 at the cell surface.

**Fig. 3:**
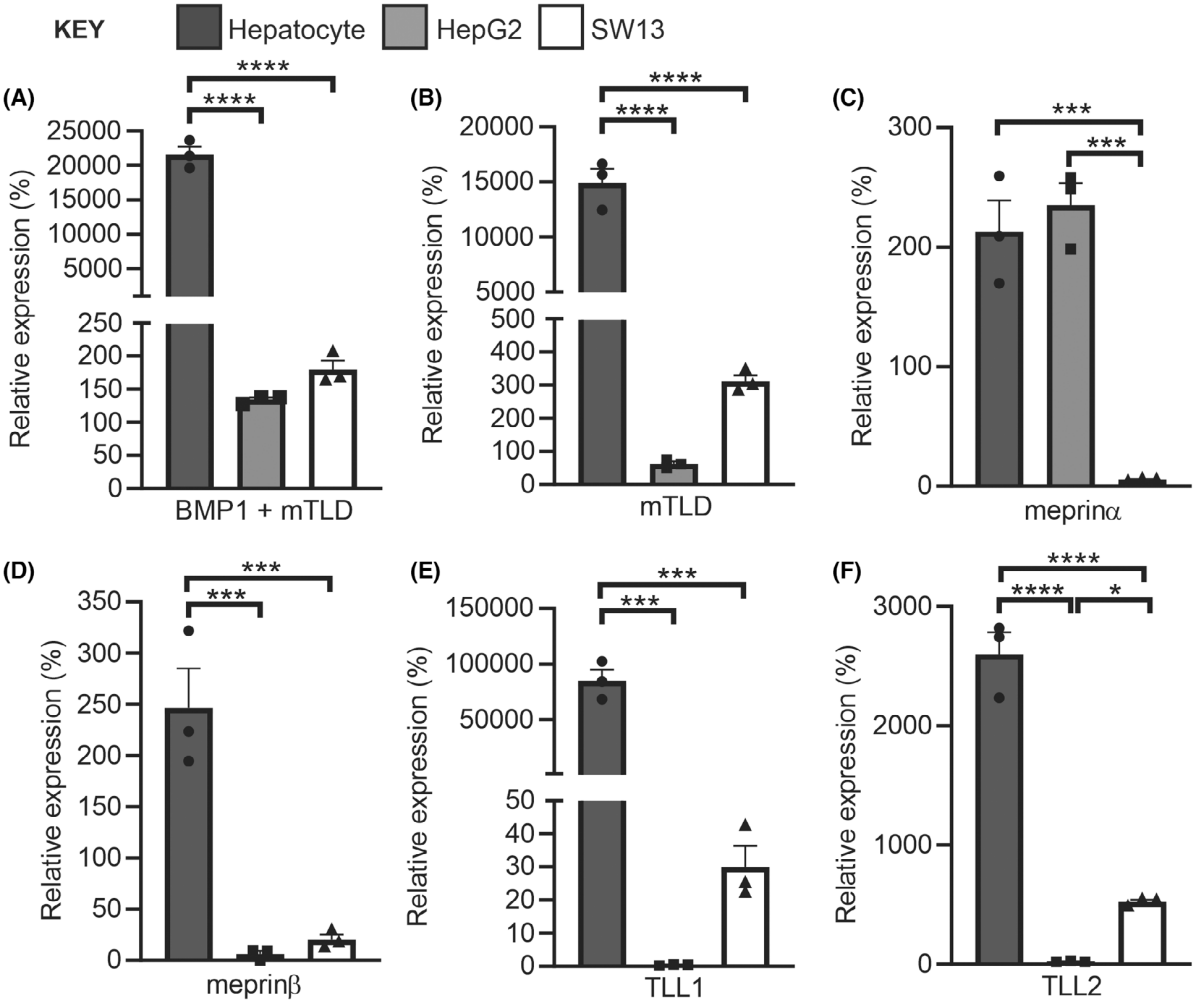
The expression profile of the astacin proteases in human cells. RNA samples from hepatocytes, HepG2, SW13 and HEK293 cells were analysed for mRNA expression of BMP1, mTLD, meprin α, meprin β, TLL1 and TLL2 using quantitative real‐time RT‐PCR. The mRNA levels were quantified relative to RPL13A. The expression of (A) BMP1 + mTLD, (B) mTLD, (C) meprin α, (D) meprin β, (E) TLL1 and (F) TLL2 are shown relative to the expression of each astacin protease in HEK293 cells. Data were analysed by using an ordinary one‐way ANOVA test with Tukey's multiple comparison test to compare between groups. *P < 0.05, ***P < 0.001, ****P < 0.0001, *n* = 3 experimental repeats for all analyses.

Next, the expression profile of members of the astacin family in the hepatocytes was compared with that in the HepG2 and SW13 cells (Fig. 3A–F). All six astacin family members were expressed in the hepatocytes, albeit at different levels of expression. As the BMP1 transcript is a shortened version of the mTLD transcript from the same gene, it is not possible to measure the BMP1 transcript alone (Fig. 3A). Meprin α was expressed in the HepG2 cells, although meprin β mRNA was barely detectable, in agreement with a previous analysis [10]. Similarly, TLL1 and TLL2 were essentially undetectable in the HepG2 cells. All of the astacin proteases were expressed in the SW13 cells, although meprin α mRNA was minimally detected. Together these data indicate that in human primary hepatocytes LDLR is subject to proteolytic cleavage within its ligand‐binding domain to generate the CTF and that the hepatocytes express all six of the astacins.

### Inhibition of BMP1 but not meprin α or β reduces LDLR proteolytic cleavage

**Fig. 4:**
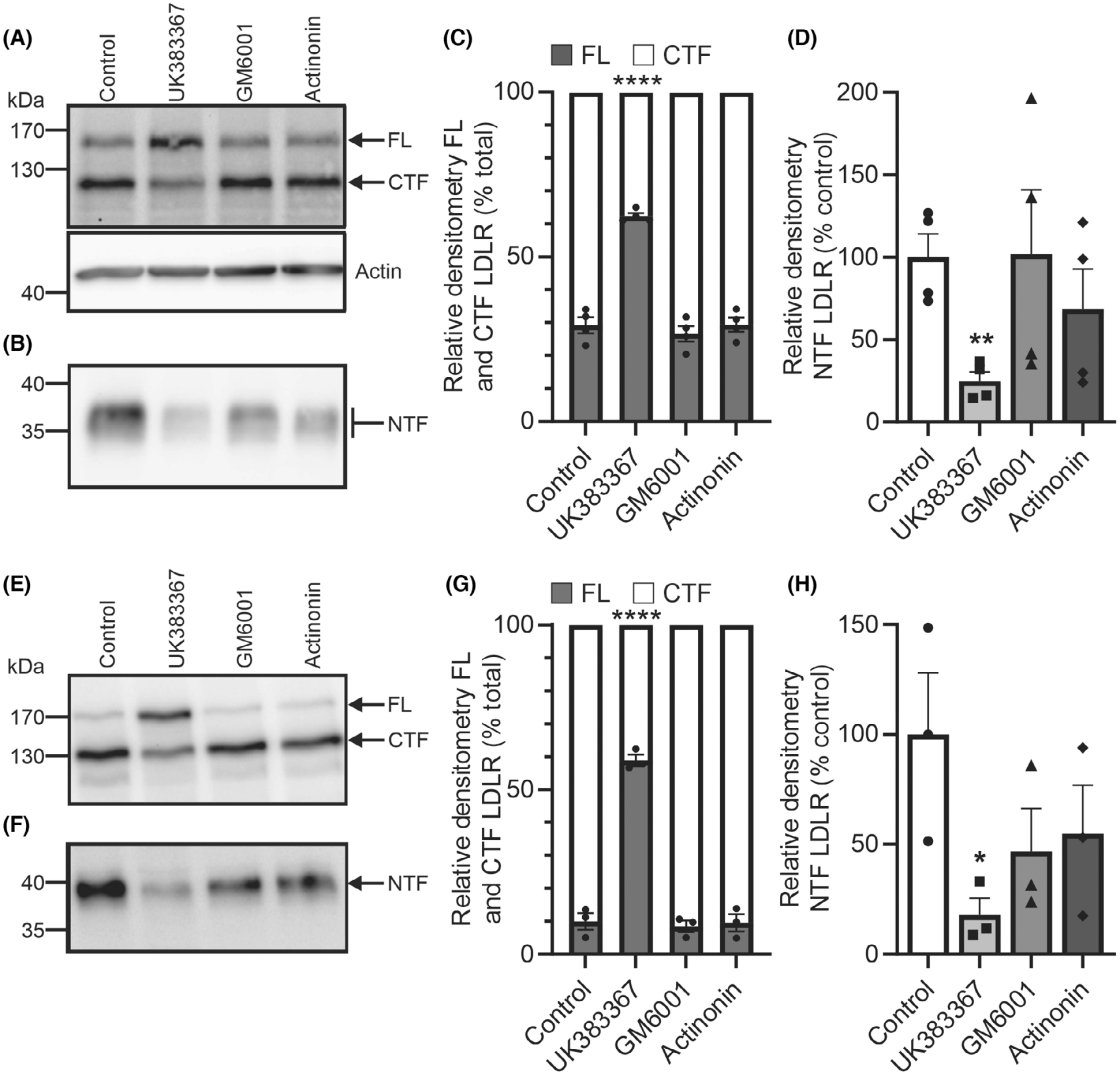
Pharmacological inhibition of BMP1, but not meprin α or meprin β, reduces cleavage of LDLR in HepG2 cells and primary hepatocytes. Human primary hepatocytes and HepG2 cells were cultured to confluency before treatment with the inhibitors UK383367 (10 μm), GM6001 (10 μm) or Actinonin (10 μm) for 24 h in OptiMEM. The cells and conditioned media were then harvested; lysates were prepared and the conditioned media concentrated. The LDLR profile in HepG2 cells was compared by western blot of (A) cell lysates and (B) conditioned media. Actin was used as a loading control. (C) The relative amounts of FL LDLR and the CTF were quantified by densitometry and expressed as a percentage of the total LDLR. (D) The relative amounts of the NTF were quantified by densitometry and are expressed as a percentage of the control (uninhibited) NTF level. The LDLR profile in primary hepatocytes was compared by western blot of (E) cell lysates and (F) conditioned media. (G) The relative amounts of FL LDLR and CTF were quantified by densitometry and expressed as a percentage of the total LDLR. (H) The relative amounts of NTF were quantified by densitometry and are expressed as a percentage of the control (uninhibited) NTF level. Data are shown as mean ± SEM. FL LDLR to CTF comparisons were made using a two‐way ANOVA with Dunnet's multiple comparison test to compare the data from each inhibitor to the uninhibited control. NTF comparisons for HepG2 data were made using a Welch's ANOVA with unpaired *t*‐tests with Welch's correction (due to unequal standard deviations) to compare the data from each inhibitor to the control. NTF comparisons for hepatocyte data were made using an ordinary ANOVA with multiple comparisons made using a Fisher's LSD test to compare the data from each inhibitor to the control. *P < 0.05, **P < 0.01, ****P < 0.0001, *n* = 4 experimental repeats for HepG2, *n* = 3 experimental repeats for hepatocytes using one cell preparation.

To investigate further the contribution of BMP1, meprin α and meprin β to the proteolytic cleavage of LDLR in the HepG2 cells and primary hepatocytes, we utilised three small molecule inhibitors that differentially inhibit one or more of these proteases (Table 1). UK383367 is more potent on BMP1 than meprin α and does not inhibit meprin β, GM6001 is inactive on BMP1 and meprin β but inhibits meprin α, and actinonin is a good inhibitor of meprin α, displays some activity towards meprin β but is inactive on BMP1 [11]. In HepG2 cells, UK383367 but neither GM6001 nor actinonin inhibited the production of the CTF of LDLR in the cell lysate (Fig. 4A,C). A similar pattern of inhibition was observed in the primary hepatocytes, with only UK383367 inhibiting the production of the CTF of LDLR (Fig. 4E,G). For completeness, we also assessed the effect of the inhibitors on the NTF of LDLR that is released into the cell medium, although it should be noted that, unlike the CTF, the NTF is relatively unstable and its levels are more variable [3]. Only UK383367 significantly inhibited the production of the NTF in both the HepG2 cells (Fig. 4B,D) and the hepatocytes (Fig. 4F,H). These data indicate that in HepG2 cells and in human hepatocytes pharmacological inhibition of BMP1, but not meprin α or meprin β, prevents proteolytic cleavage of LDLR in its ligand‐binding domain.

**Table 1:** Half‐maximal inhibitory concentrations of inhibitors for BMP1, meprin α and meprin β. Data from [11].

Inhibitor	IC_50_ (nm)		
	BMP1	Meprin α	Meprin β
UK383367	55	249	> 3000
GM6001	> 100 000	185	> 10 000
Actinonin	> 10 000	55	744

### Genetic knockdown of BMP1 but not other astacins reduces the proteolytic cleavage of LDLR

**Fig. 5:**
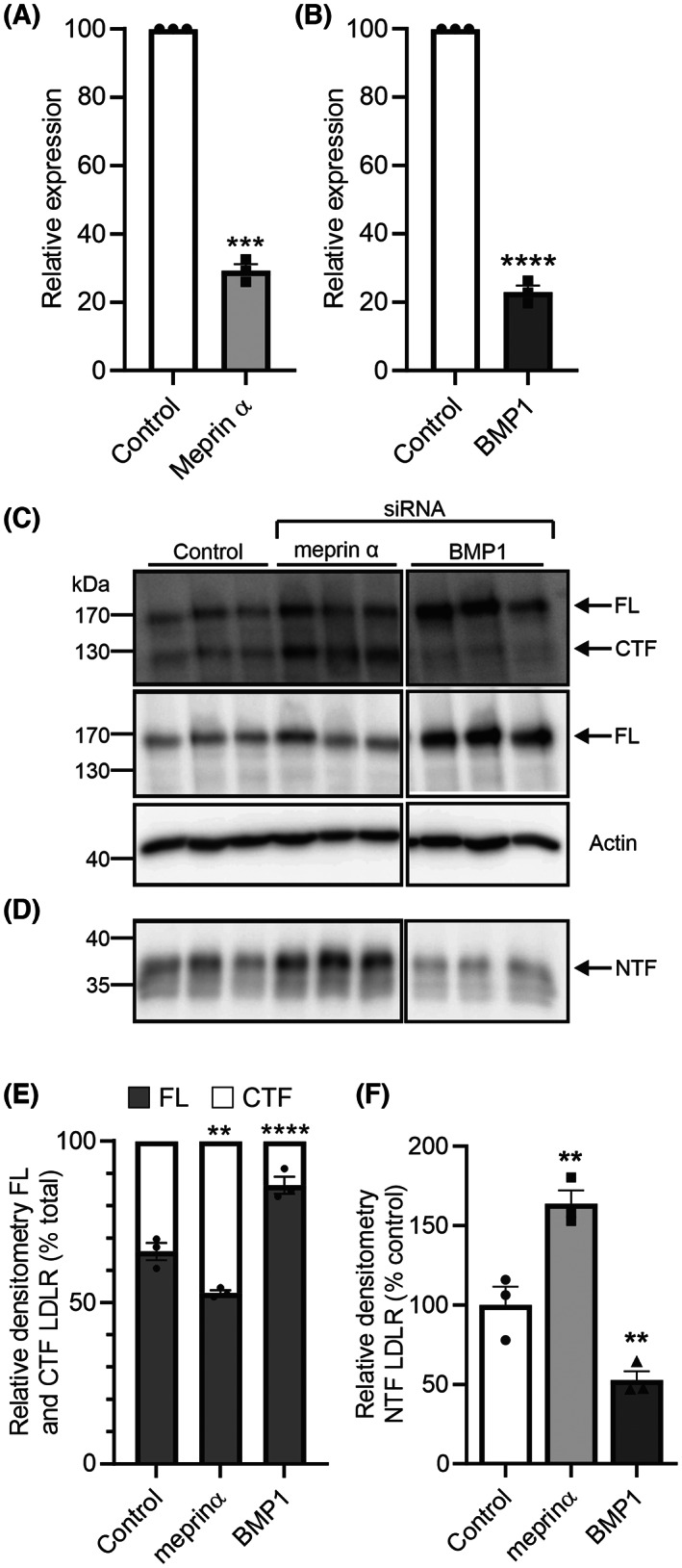
siRNA knockdown of BMP1, but not meprin α, reduces LDLR cleavage in HepG2 cells. HepG2 cells were cultured to 70% confluency and treated with either non‐targeting siRNA or siRNA (50 nm) for meprin α or BMP1 for 48 h before incubation for a further 24 h in OptiMEM. The cells and conditioned media were then harvested; lysates and RNA samples were prepared and the conditioned media was concentrated. Knockdown of meprin α (A) or BMP1 (B) was confirmed by mRNA analysis using real‐time quantitative PCR. The mRNA expression for each gene was calculated relative to RPL13A and the expression shown relative to control. The LDLR profile in HepG2 cells was compared by western blot of (C) cell lysates and (D) conditioned media (samples were analysed on the same blot with irrelevant lanes between the meprin α and BMP1 samples removed for clarity). Actin was used as a loading control. (E) The relative amounts of FL LDLR and CTF were quantified by densitometry and expressed as a percentage of the total LDLR. (F) The relative amounts of NTF were quantified by densitometry and are expressed as a percentage of the control (uninhibited) NTF level. Data are shown as mean ± SEM. Comparisons for mRNA expression were made using an unpaired *t*‐test with Welch's correction. FL LDLR to CTF comparisons were made using a two‐way ANOVA with Dunnett's multiple comparison test to compare the data from each inhibitor to the control. NTF comparisons were made using an ordinary one‐way ANOVA with Fisher's LSD test to compare the data from each inhibitor to the control. **P < 0.01, ***P < 0.001, ****P < 0.0001, *n* = 3 experimental repeats for all analyses.

**Fig. 6:**
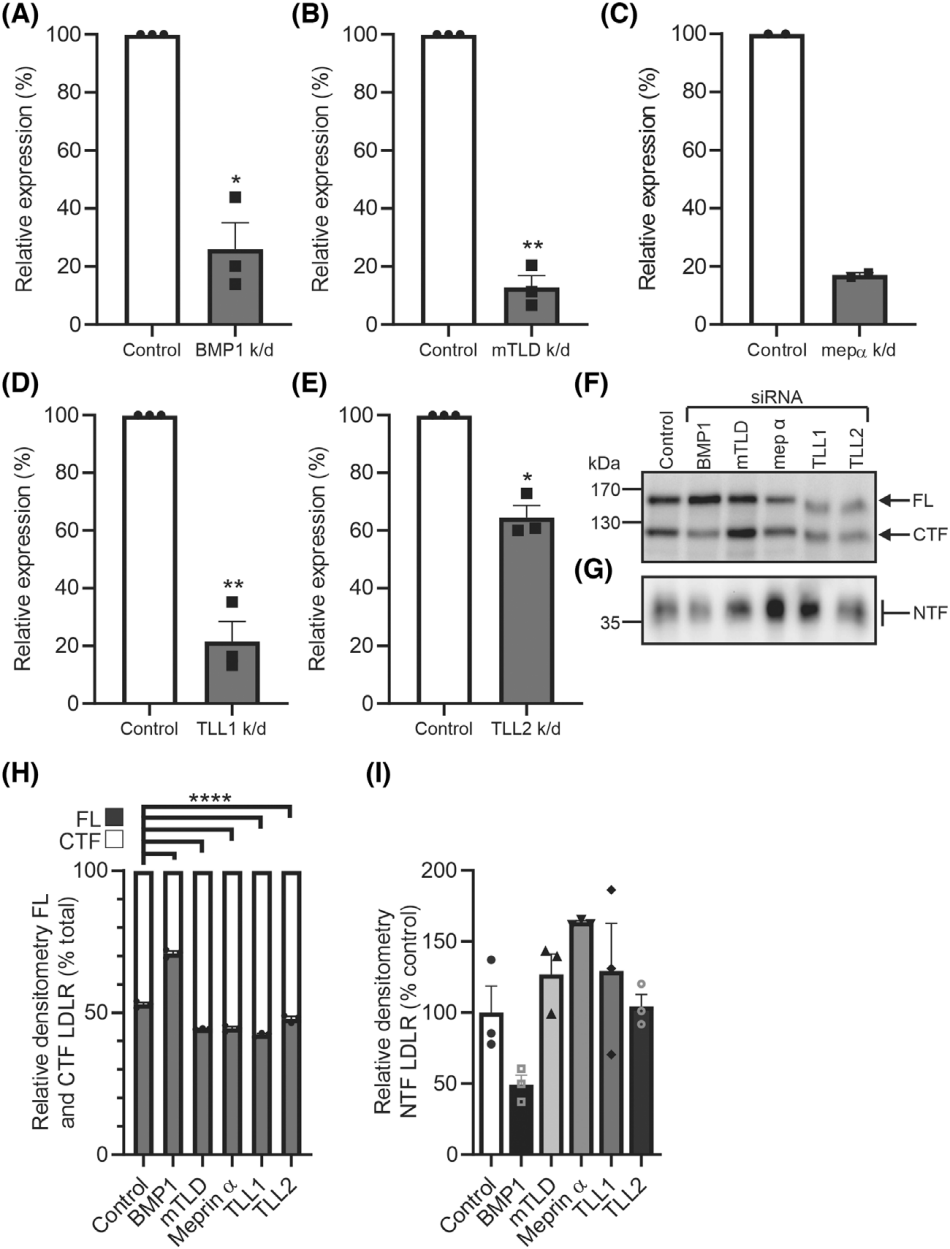
siRNA knockdown of BMP1, but not other astacins, prevents LDLR cleavage in human hepatocytes. Human hepatocytes were cultured to 70% confluency and treated with either non‐targeting siRNA or siRNA (50 nm) for BMP1, mTLD, meprin α, TLL1 or TLL2 for 24 h before incubation for a further 24 h in OptiMEM. The cells and conditioned media were then harvested; lysates and RNA samples were prepared and the conditioned media was concentrated. (A–E) Knockdown was confirmed by mRNA analysis using real‐time quantitative PCR. The mRNA expression for each gene was calculated relative to RPL13A and the expression shown relative to control. The LDLR profile in hepatocytes was compared by western blot of (F) cell lysates and (G) conditioned media. Total protein staining by amido black was used as a loading control. The relative amounts of (H) FL LDLR and CTF were quantified by densitometry and expressed as a percentage of the total LDLR. (I) The relative amounts of NTF were quantified by densitometry and are expressed as a percentage of the control (non‐targeting siRNA) NTF level. Data are shown as mean ± SEM, except for in (C) where data are shown as median and range. Comparisons for mRNA expression were made using an unpaired *t*‐test with Welch's correction. FL LDLR to CTF comparisons were made using a two‐way ANOVA with Dunnett's multiple comparison test to compare the data from each knockdown to the control. NTF comparisons were made using an ordinary ANOVA test with multiple comparisons made using a Fisher's LSD comparison test to compare the data from each knockdown to the control. *P < 0.05, **P < 0.01, ****P < 0.0001, *n* = 3 experimental repeats from one hepatocyte preparation for all analyses, except in (C) where *n* = 2.

To confirm the pharmacological inhibition data, siRNA was used to selectively knockdown the expression of the astacins. Initially, meprin α and BMP1 were knocked down in the HepG2 cells (Fig. 5). BMP1 and meprin α were knocked down by 78% and 71% by their respective siRNA (Fig. 5A,B). However, only knockdown of BMP1 reduced the formation of the NTF and CTF of LDLR (Fig. 5C–F). Knockdown of meprin α resulted in an increase in the amount of CTF and NTF, likely as a result of increasing the amount of full length LDLR through an unknown mechanism (Fig. 5C–F). Next, all of the astacins, except for meprin β whose expression was low in the hepatocytes (Fig. 3E), were knocked down using siRNA in the human hepatocytes (Fig. 6). BMP1, mTLD, meprin α and TLL1 were all knocked down by > 70%, with the maximum knock down of TLL2 reaching 35%. Only knockdown of BMP1 increased the ratio of CTF to full length LDLR in the cell lysate and the amount of NTF in the cell media (Fig. 6F–I). Knockdown of meprin α, mTLD, TLL1 or TLL2 did not increase the ratio of CTF to full length LDLR or decrease the amount of NTF (Fig. 6F–I). Together with the pharmacological inhibition data, these genetic knockdown data confirm that BMP1, but not other astacin family members, is responsible for the proteolytic cleavage of LDLR within its ligand‐binding domain in both human hepatocytes and HepG2 cells.

### A double point mutation in mouse LDLR enables proteolytic cleavage by BMP1

**Fig. 7:**
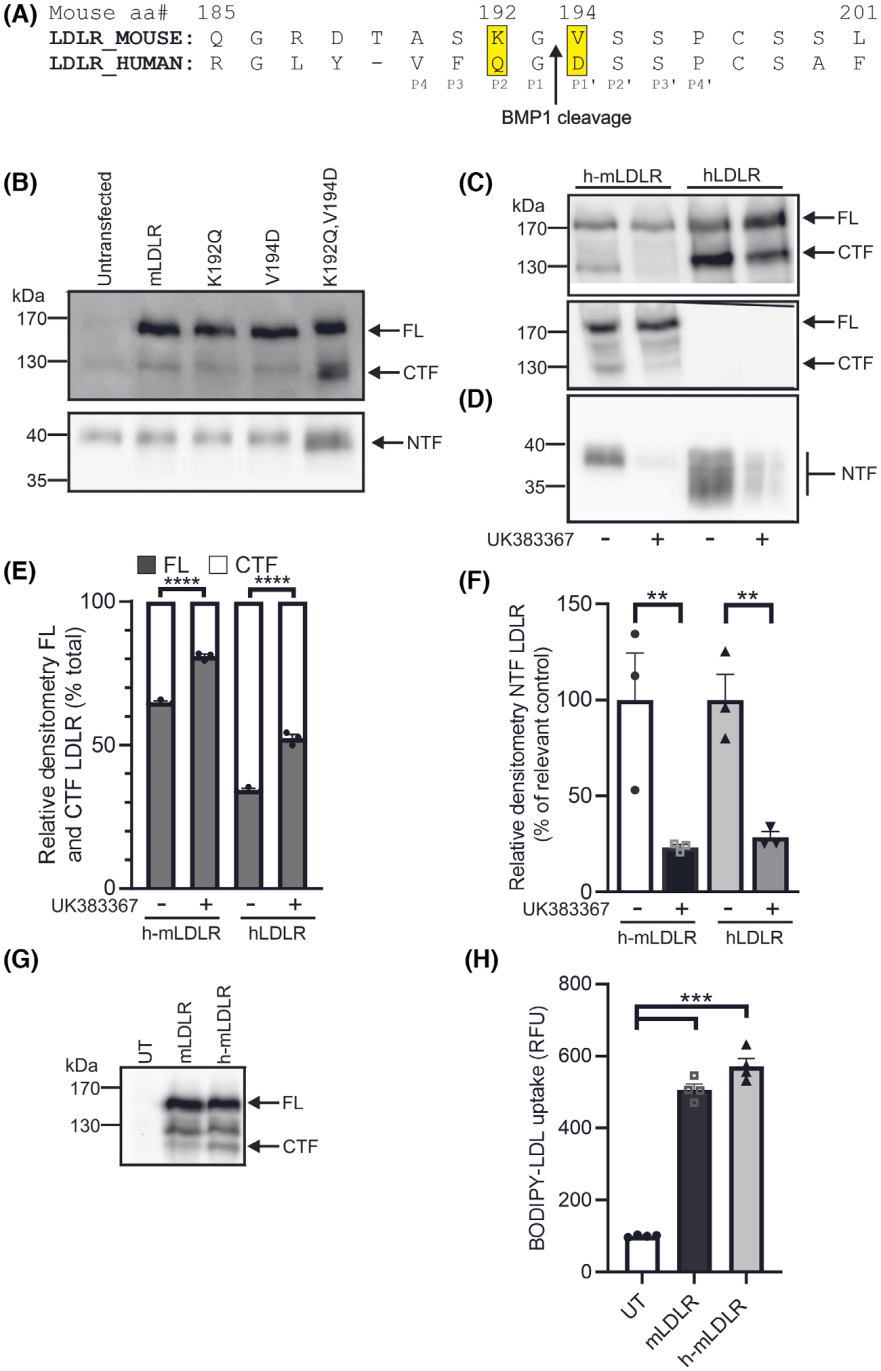
‘Humanised’ mouse LDLR is proteolytically cleaved by BMP1 and internalises LDL‐C. (A) Comparison of the mouse and human LDLR sequences with the two key residues in the P1′ and P2 positions that differ between them highlighted in yellow. The single point mutations K192Q and V194D, as well as the double mutation K192Q, V194D were made to the mouse LDLR sequence. These constructs were sequence verified before being transfected into HepG2 cells. The cells were then cultured to confluency before incubation for 24 h in OptiMEM. The cells and conditioned media were harvested; lysates were prepared and the conditioned media was concentrated. The LDLR profile in HepG2 cells expressing either mouse LDLR (mLDLR), mouse LDLR K192Q, mouse LDLR V194D or mouse LDLR K192Q, V194D (h‐mLDLR) was compared by western blot of cell lysates using AF2148 to show FL and CTF and of conditioned media using Ab14056 to show the NTF (B). HepG2 cells were transfected with either hLDLR or h‐mLDLR before incubation for 24 h in OptiMEM without or with the BMP1 inhibitor UK383367 (10 μm). The cells and conditioned media were harvested; lysates were prepared and the conditioned media was concentrated. The LDLR profile in HepG2 cells expressing either h‐mLDLR or hLDLR treated without or with UK383367 (10 μm) was compared by western blot of cell lysates using both a human LDLR antibody (AF2148) (C‐upper panel) and a mouse LDLR antibody (AF2255; C‐lower panel) and of conditioned media (D). The relative levels of FL LDLR and CTF were quantified by densitometry and expressed as a percentage of the total LDLR (E). The relative levels of NTF were quantified by densitometry and are expressed as a percentage of the relevant control (F). mLDLR and h‐mLDLR constructs were transfected into CHO‐A7 cells, which lack endogenous LDLR expression. The cells were cultured to confluency before incubation for 24 h in OptiMEM. The cells were then harvested; lysates were prepared and the LDLR profile compared by western blot using a mouse LDLR antibody (AF2255) (G). Transfected CHO‐A7 cells were cultured in 96‐well plate format and media spiked with BODIPY‐LDL to evaluate LDL uptake into transfected cells (H). Data are shown as mean ± SEM. FL LDLR and CTF comparisons were made using a two‐way ANOVA with Dunnett's multiple comparison test to compare each treated group with its control. NTF comparisons were made using an ordinary ANOVA with pre‐selected comparisons made using a Fisher's LSD test to compare the inhibitor treated samples with the relevant control. BODIPY‐LDL uptake comparisons were made using a Welch's ANOVA with comparisons made using a Dunnett T3 test to compare each group against control. **P < 0.01, ***P < 0.001, ****P < 0.0001, *n* = 3 experimental repeats for HepG2 data in (C–F), *n* = 4 experimental repeats for BODIPY‐LDL analysis in (G, H). UT, untransfected.

Previously we reported that mLDLR was not cleaved by BMP1 [3], likely due to the Asp (D193) in the linker region between the fourth and fifth ligand repeats in hLDLR being substituted with Val (V194) in mLDLR (Fig. 7A; note hLDLR is one amino acid shorter than mLDLR in this region due to an additional residue at position 190 in mLDLR). Residues further away from the scissile peptide bond can impact on the cleavage of substrates by BMP1 and of note is the presence of Gln (Q191) at the P2 position in hLDLR which is replaced with Lys (K192) in mLDLR (Fig. 7A). Comparison of multiple substrates of BMP1 indicates that Gln at the P2 position is well‐tolerated but that Lys has yet to be found in this position in any BMP1 substrate [12]. In contrast, at position P3 both Phe and Ser have been reported in BMP1 substrates, and similarly Ala and Val at position P4 [12]. To explore this sequence requirement and in an attempt to make mouse LDLR susceptible to cleavage by BMP1, we generated three mutants of mLDLR, one in which Val194 was changed to Asp (V194D), one in which Lys192 was changed to Gln (K192Q) and one in which both Lys192 was changed to Gln and Val194 was changed to Asp (K192Q/V194D; Fig. 7A). The mutated mLDLR constructs were expressed in HepG2 cells, alongside wild‐type mLDLR (Fig. 7B). Neither of the single point mutation (K192Q or V194D) constructs were cleaved to generate the CTF and NTF. However, the double point mutation (K192Q/V194D) construct was cleaved to produce the CTF and NTF (Fig. 7B). This h‐mLDLR double point mutation construct was then expressed alongside hLDLR in the HepG2 cells and the effect of the BMP1 inhibitor on the cleavage of h‐mLDLR assessed(Fig. 7C,D). The production of both the CTF and the NTF was reduced in the presence of the BMP1 inhibitor in the cells expressing h‐mLDLR, similarly to the effect on cleavage of hLDLR (Fig. 7E,F).

To ensure that the point mutations in h‐mLDLR that render it susceptible to cleavage by BMP1 do not impact on the functionality of the receptor, h‐mLDLR was expressed alongside mLDLR in CHO‐A7 cells which lack endogenous LDLR [13] and the uptake of BODIPY LDL assessed. Both constructs of LDLR were expressed in the CHO‐A7 cells with h‐mLDLR being cleaved to produce the CTF (Fig. 7G). In the cells expressing h‐mLDLR, BODIPY‐LDL internalisation was increased above that in the untransfected cells, similar to that seen in the cells expressing the mLDLR construct (Fig. 7H), indicating that the h‐mLDLR was able to bind and internalise LDL. Together these data indicate that the double point mutation in the linker region between LA repeats 4 and 5 in mLDLR renders the receptor susceptible to cleavage by BMP1 and that the resulting h‐mLDLR construct is functional in internalising LDL.

## Discussion

In order to maintain cellular cholesterol homeostasis, the function of LDLR is tightly controlled [2]. Regulation of cell surface LDLR can be mediated through selective proteolytic cleavage. Recently, membrane type 1 matrix metalloproteinase was reported to promote the shedding of the entire ectodomain of LDLR and accelerate the development of atherosclerosis [14]. We and others have shown that BMP1 cleaves LDLR within its ligand binding‐domain regulating the binding and cellular uptake of LDL‐C [3, 4]. Here, we show that although BMP1 is closely related both structurally and catalytically to other members of the astacin metalloprotease family, only BMP1 contributes significantly to LDLR proteolysis in human hepatocytes and HepG2 cells. Furthermore, although mLDLR is not cleaved by BMP1, using knowledge of the substrate specificity of BMP1, we have explored the minimum number of mutations that renders mLDLR susceptible to cleavage by BMP1. The resulting h‐mLDLR construct was effectively cleaved by BMP1 in its ligand‐binding domain and internalised LDL‐C.

All astacin family members have a marked preference for Asp at the P1′ site relative to the peptide bond being cleaved [9] and can thus be considered as candidates to cleave LDLR at the Gly‐Asp site in the linker region between LA repeats 4 and 5. To exploit the cleavage of LDLR by BMP1 as a potential strategy to treat hypercholesterolemia, it is important to know whether other astacins are also capable of cleaving LDLR within its ligand‐binding domain. To investigate the contribution of other astacin proteases to the proteolysis of LDLR we first considered their cellular expression. In HepG2 cells, in which LDLR is proteolytically cleaved within its ligand‐binding domain, meprin β, TLL1 and TLL2 were either not expressed or minimally expressed, arguing against these proteases contributing significantly to LDLR cleavage. This is supported by the data from the SW13 cells, in which LDLR is not subject to proteolytic cleavage in its ligand‐binding domain [3], but in which meprin β, TLL1 and TLL2 are all expressed. The observations that TLL1 is not expressed in either mouse or human liver, and TLL2 is expressed at only a low level in human liver [15] (D. Greenspan, personal communication), further argues against these two proteases being involved in LDLR proteolysis, at least in the liver. In contrast, BMP1/mTLD are expressed in mouse and human liver [16] (D. Greenspan, personal communication). We note that the experiments performed in the current study utilised the HepG2 cell line and primary hepatocytes rather than human liver, although we have previously reported the cleavage of LDLR in human liver samples [3].

As the meprins are transmembrane proteins (Fig. 1), they would possibly appear as better candidates than the soluble BMP1 to cleave the membrane‐bound LDLR [17]. Using a panel of inhibitors with different selectivity towards BMP1, meprin α and meprin β, along with siRNA knockdown, we show that neither meprin contributes to the cleavage of LDLR in human hepatocytes or HepG2 cells. BMP1 clearly has the capacity to directly cleave membrane‐bound LDLR, as evidenced from our previous work showing that recombinant BMP1 can cleave recombinant LDLR in a cell‐free assay and that the action of BMP1 on LDLR is not dependent on prior shedding of the extracellular domain as the CTF is still transmembrane [3]. Whether other membrane‐bound proteins are also substrates for BMP1 awaits investigation.

In addition to its catalytic domain, BMP1 has several non‐catalytic, protein–protein interaction domains (Fig. 1). The CUB domains have a Ca^2+^‐binding site that mediates ionic interactions between protein partners, similar to that described for the EGF‐like domain in LDLR [18]. These non‐catalytic domains on BMP1 and other astacin family members are important for substrate recognition and for controlling and restricting their proteolytic activity [19, 20, 21]. The metalloproteinase and CUB2 domains of BMP1 are absolutely required for cleavage of procollagen, and the metalloprotease and CUB1 domains are the minimal structure required for cleaving chordin [19]. There is the possibility that one or more of its non‐catalytic domains is also required for the interaction with LDLR, and that these interactions are responsible for the ability of BMP1, but not other astacin proteases, to cleave LDLR in its ligand‐binding domain.

Our data showing that LDLR is cleaved by BMP1 in human primary hepatocytes adds further weight to this proteolytic cleavage event being of physiological relevance. The CTF of LDLR resulting from BMP1 cleavage has been identified in extracts from multiple human liver samples and human adrenal gland and the NTF was detected in human urine, likely due to clearance from plasma by the kidneys [3, 22]. However, as mouse LDLR is not cleaved by BMP1 due to the critical Asp193 being replaced by Val, studies in mice expressing wild‐type LDLR would not reveal whether cleavage of the receptor by BMP1 has an impact on plasma LDL‐C metabolism. As the first step to overcome this issue, we explored the minimum sequence changes required to render mLDLR susceptible to cleavage by BMP1. Mutation of both Lys192 and Val194 at the P2 and P1′ positions, respectively, were required to allow mLDLR to be cleaved by BMP1. The resulting h‐mLDLR construct was capable of internalising LDL‐C when expressed in cells, suggesting that this h‐mLDLR construct could be used as the basis for generating a transgenic mouse model in which the contribution of the BMP1 cleavage of LDLR to plasma LDL‐C metabolism could be explored *in vivo*.

In conclusion, we show that LDLR is proteolytically cleaved by BMP1 in human hepatocytes and that BMP1 is the only member of the astacin family responsible for cleaving LDLR in its ligand binding region. Incorporation of a double point mutation in the linker region between LA repeats 4 and 5 in mLDLR renders it susceptible to proteolytic cleavage by BMP1, and when expressed in cells, the resulting h‐mLDLR construct internalises LDL‐C. Further insight into the role of BMP1 in LDLR metabolism, including the development of a humanised LDLR mouse model, will extend our understanding of the biological mechanisms controlling plasma LDL‐C and could lead to new therapeutic targets for the treatment of hypercholesterolaemia.

## Author contributions

KABK and NMH designed and coordinated the study. KF, HA and KABK performed experiments. KABK and NMH analysed and interpreted the data, and wrote the paper. All authors reviewed the results, critically revised the manuscript and approved the final version.

## Data Availability

The data supporting the findings reported in this paper are openly available from the University of Manchester FigShare repository at https://doi.org/10.48420/22821425.v1.
